# Artemis as Predictive Biomarker of Responsiveness to Preoperative Chemoradiotherapy in Patients with Locally Advanced Rectal Cancer

**DOI:** 10.3390/curroncol31010037

**Published:** 2024-01-18

**Authors:** Hai Liu, Runying Huang, Jingjing Shan, Xuyun Xie, Chongwei Wang, Peng Hu, Xiaonan Sun

**Affiliations:** 1 Department of Radiation Oncology, Sir Run Run Shaw Hospital, School of Medicine, Zhejiang University, Hangzhou 310016, China; 3311005@zju.edu.cn (H.L.); hryleo@163.com (R.H.); 21318149@zju.edu.cn (J.S.); 3413007@zju.edu.cn (X.X.); 2 Department of Pathology, Sir Run Run Shaw Hospital, School of Medicine, Zhejiang University, Hangzhou 310016, China; cadillac_luck@zju.edu.cn; 3 Department of Radiology, Sir Run Run Shaw Hospital, School of Medicine, Zhejiang University, Hangzhou 310016, China; ph198252@zju.edu.cn

**Keywords:** Artemis, preoperative chemoradiotherapy, radioresistance, rectal cancer

## Abstract

The aim of this study was to identify Artemis as a predictive biomarker for guiding preoperative chemoradiotherapy in locally advanced rectal cancer. The resection specimens were collected from 50 patients with rectal cancer who underwent preoperative chemoradiotherapy. Artemis expression in biopsy tissues was evaluated using immunohistochemical staining according to the percentage of positively stained cells combined with staining intensity. Among the 50 patients, 36 (72%) had a weakly positive Artemis protein expression, 10 (20%) had a moderately positive expression, and 4 (8%) showed a strongly positive expression. The criteria of magnetic resonance imaging tumor regression grade (mrTRG) and pathological rectal cancer regression grade (RCRG) were used to assess the tumor response to chemoradiotherapy. Correlation analysis shows that there is a significant negative correlation between high Artemis immunoscore and treatment response (*r* = −0.532, *p* < 0.001). The results imply that high Artemis expression was associated with poor treatment response. Our study suggested a potential role of Artemis as a predictive biomarker of the tumor response to preoperative chemoradiotherapy in patients with locally advanced rectal cancer.

## 1. Introduction

Colorectal cancer is the third-most common malignant cancer and the second-leading cause of cancer mortality globally [1]. The standard treatment for locally advanced rectal cancer (LARC) is preoperative chemoradiotherapy followed by surgical resection with total mesorectal excision (TME) [2,3]. Especially for patients with mid-low rectal cancer and T3 or T4 stage, preoperative chemoradiotherapy can effectively reduce the tumor volume, increase sphincter preservation, decrease local recurrence, and have long-term survival benefits [4]. However, the therapeutic effect of preoperative chemoradiotherapy on patients with rectal cancer varies significantly among individuals, with only a few patients (9–37%) achieving a pathological complete response (pCR) after treatment [5]. Patients who achieve pCR after preoperative chemoradiotherapy have better outcomes [6,7]. In recent years, the organ-preserving treatment approach, known as the watch and wait (W&W) strategy, has gained wide recommendation. This strategy involves omitting surgery in selected patients who have achieved a clinical complete response after neoadjuvant chemoradiotherapy [8]. It is an attractive option as it helps to avoid the risks associated with surgery, such as morbidity and mortality, and improves the quality of life for patients. A large international registry-based study, the International Watch & Wait Database, has demonstrated satisfying long-term outcomes and superiority in terms of quality of life [9]. By correctly selecting patients who have shown a good response to preoperative chemoradiotherapy, the accuracy of patient selection, monitoring, and prognosis can be improved under this preservation strategy. On the other hand, due to the heterogeneity of treatment response, some patients may not respond well to preoperative chemoradiotherapy. For these patients, undergoing preoperative radiotherapy may result in a delay in surgery and lead to severe toxicities, including anastomotic leakage and perineal wound infection [10]. It is important to identify the sensitivity of the tumor to preoperative chemoradiotherapy before initiating treatment in order to avoid unnecessary treatment for nonresponding groups. Therefore, the presence of a validated biomarker that can predict the response to preoperative chemoradiotherapy becomes crucial for tailoring the treatment of rectal cancer patients on an individual basis.

Currently, many molecular biomarkers have been examined as potential predictors of the rectal cancer response to preoperative radiotherapy. However, no sufficient evidence for any of these biomarkers has been reported to date to be introduced into clinical practice [11]. The therapeutic effect of irradiation (IR) largely results from double-stranded DNA breaks (DSBs), with nonhomologous end-joining (NHEJ) playing a major role in IR-induced DSB repair in human cells throughout the cell cycle [12]. When double-stranded DNA breaks (DSBs) occur due to ionizing radiation, the NHEJ process begins with Ku70/80 binding to the double-stranded DNA ends at the DSB site. Subsequently, the nuclease complex (Artemis:DNA-PKcs complex), DNA polymerases, and the ligase complex (XLF:XRCC4:DNA ligase IV and PAXX) come into action until both strands are ligated [13,14]. Artemis (DNA crosslink repair 1C, DCLRE1C) is an endonucleolytic enzyme that is a component of the NHEJ DNA DSB repair pathway. Artemis is the major nuclease used to trim broken DNA ends, making them suitable for ligation by DNA ligase. Artemis is also responsible for opening DNA hairpins during V(D)J recombination. Artemis functions in V(D)J recombination and NHEJ as a hairpin and 5′ and 3′ overhang endonuclease, which can remove lesions or secondary structures, thereby inhibiting end resection and precluding the completion of NHEJ [15,16]. The role of Artemis in DNA repair also includes regulating the cell cycle and maintaining normal telomere function [17,18]. Extensive research has demonstrated the crucial role of Artemis in radiation-induced DNA damage. Cells deficient in Artemis exhibited hypersensitivity to radiation [17], whereas the overexpression of Artemis provided significant radioprotection against both high and low linear energy transfer (LET) radiation [19]. Thus, the expression level of Artemis is a critical determinant of radiosensitivity.

Our previous study found that the knockdown of Artemis in human colorectal cancer cells enhanced the sensitivity to DNA-damaging agents, including radiation. Our preliminary study also found that more than half of the patients with rectal cancer had a moderate or high expression of Artemis protein [20]. However, whether Artemis is a useful biomarker for guiding preoperative radiotherapy in LARC remains unclear. In this study, we enrolled 50 patients with LARC treated with fractionated preoperative radiotherapy and radical resection in Sir Run Run Shaw Hospital. Artemis expression was detected in pretreatment colonoscopy biopsy specimens. The tumor regression of the primary tumor was determined in pelvic MRI after chemoradiotherapy and the resection of specimens. Finally, we explored whether Artemis was a predictive marker of the tumor response to preoperative chemoradiotherapy.

## 2. Materials and Methods

### 2.1. Patients and Neoadjuvant Therapy

A total of 50 patients with LARC were enrolled at Sir Run Run Shaw Hospital of Zhejiang University between January 2009 and July 2013. All patients were diagnosed with adenocarcinoma by colonoscopy and biopsy. Staging was conducted using endoscopic ultrasonography, pelvic MRI, and total-body computed tomography (CT). All patients received preoperative chemoradiotherapy involving a total dose of 45.0–50.4 Gy of IR delivered to the pelvic region in 25–28 fractions with concurrent capecitabine chemotherapy. Radical surgery (low anterior resection or abdominoperineal resection) was performed in 4–14 weeks after completing preoperative chemoradiotherapy. Biopsy and resection specimens were collected and analyzed retrospectively. This study was approved by the local scientific and research ethics committees of Sir Run Run Shaw Hospital.

### 2.2. MRI Examination and MRI Tumor Regression Grade

Pelvic MRI scans were conducted using a GE Signa 1.5T Excite HD MRI Scanner equipped with a pelvic phased-array coil before and after chemoradiotherapy. T1-weighted images, T2-weighted sequences with and without fat suppression, and dynamic sequences of T1 high-resolution isotropic volume excitation were acquired in the axial and sagittal planes. The MRI tumor regression grade (mrTRG) was used to assess tumor regression on imaging after chemoradiotherapy [21]. On axial T2-weighted MRIs obtained before and after chemo-radiotherapy, primary tumor volumes were calculated by prescribing contours to calculate tumor cross-sectional areas on each section and then summing the sections to calculate the volume. The degree of primary tumor volume reduction determined the regression grade of the tumor. The characteristics of each grade were as follows: mrTRG 1 (complete radiologic response), no evidence of tumor on the MRI after treatment; mrTRG 2 (good response), dense (>75%) fibrosis with no obvious residual tumor; mrTRG 3 (moderate response), >50% fibrosis or mucin with a minority of visible tumors; mrTRG 4 (slight response), <50% fibrosis or mucin with a majority of visible tumors; and mrTRG 5 (no response), no post-treatment changes (same as before treatment).

Two radiologists blinded to the clinical and pathological characteristics associated with the specimens performed the mrTRG evaluations.

### 2.3. Pathological Response Evaluation

Two experienced pathologists divided the cases into Wheeler’s Rectal Cancer Regression Grade (RCRG) 1–3 according to the RCRG criteria to evaluate tumor response to chemoradiotherapy [22]. RCRG 1 indicated a “good” response when either the tumor was sterilized or only microscopic foci of adenocarcinoma remained. RCRG 2 reflected marked fibrosis but with macroscopic tumors still present, and RCRG 3 indicated a “poor” response with little or no fibrosis in the presence of abundant macroscopic tumors.

### 2.4. Immunohistochemistry and Evaluation of Artemis Expression

Immunohistochemical staining with Artemis antibody was performed on pathological tissues of rectal tumors in preoperative biopsy. Artemis antibody was provided by Hangzhou Huabio Biotechnology Co. (Hangzhou, China). Immunohistochemistry was carried out as described in a previous study [20]. Briefly, formalin-fixed paraffin-embedded rectal cancer tissue was mounted onto poly-l-lysine-coated slides, followed by deparaffinization and rehydration. To quench the endogenous peroxidase activity, the slides were treated with 0.3% H_2_O_2_ (in absolute methanol) for 30 min. Subsequently, 5% bovine serum albumin was applied to block nonspecific staining, and the slides were incubated overnight with primary antibodies specific to Artemis. For Artemis detection, the slides were then incubated for 1 h with biotinylated anti-rabbit IgG (Vector Laboratories Inc., Burlingame, CA, USA). Afterward, the slides were incubated with the avidin–biotin-peroxidase complex (Vector Laboratories Inc.) for 1 h, and the antibody binding was visualized using 3,3′-diaminobenzidine tetrahydrochloride. Finally, the sections were lightly counterstained with Mayer’s hematoxylin.

Two senior pathologists performed scoring independently. The evaluation of Artemis protein expression in rectal cancer tissue was based on the percentage of positively stained cells combined with staining intensity. The percentage of positive cells was scored as follows: 0 (<5%), 1 (≥5%), 2 (≥25%), 3 (≥50%), or 4 (≥75%). The staining intensity was scored as 0 (negative, no brown particles), 1 (weak, scattered with light or small brown particles), 2 (medium, large brown particles), and 3 (strong, brown, and yellow particles). The final staining score was determined by multiplying the distribution and intensity scores, resulting in a negative score: 0 (negative, −), 1–4 (weakly positive, +), 5–7 (moderately positive, ++), and 8–12 (strongly positive, +++).

### 2.5. Statistical Analysis

The data were analyzed using SPSS 25.0 statistical software (IBM, Armonk, NY, USA). The comparisons between groups were performed using the χ^2^ test or Fisher’s exact test. A Spearman nonparametric rank correlation test was used to assess the degree of correlation between the tumor response to treatment and immunoscore of Artemis expression. Kaplan–Meier analysis was used to compare the overall survival (OS). A *p* value < 0.05 (two-sided) indicated a statistically significant difference.

## 3. Results

### 3.1. Clinical Characteristics of Patients and Artemis Expression

**Figure 1:**
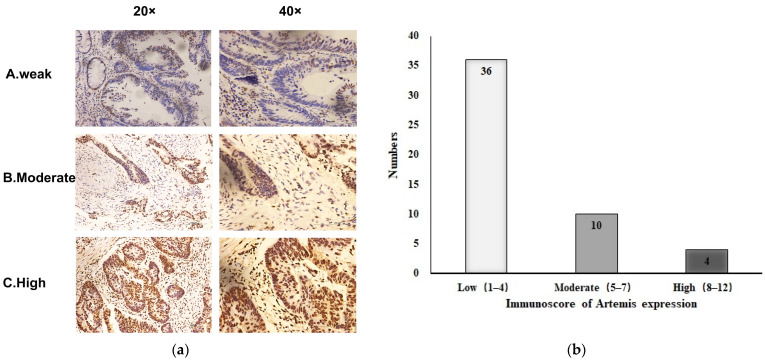
Expression of Artemis protein in biopsy specimens before neoadjuvant chemoradiotherapy. (**a**) Representative image of Artemis protein immunochemical staining in biopsy specimens from patients with rectal cancer: A, weakly positive; B, moderately positive; C, strongly positive (magnification: ×20, ×40). (**b**) Based on the immunoscore of Artemis, the expression of Artemis in 50 patients with colorectal cancer was classified as low, moderate, and high expression.

A total of 50 patients diagnosed with LARC were included in this study. All patients had undergone preoperative chemoradiotherapy and surgical treatment. We retrospectively analyzed the clinicopathological characteristics of these patients and the Artemis expression in biopsy tissues before chemoradiotherapy, as shown in Table 1. The characteristic microscopic appearance of Artemis immunostaining of the biopsy specimens is shown in Figure 1a, indicating that the Artemis protein was mainly located in the nucleus. The expression level of Artemis protein was determined using the scoring method mentioned earlier. Among the 50 patients, 36 (72%) with Artemis protein immunoscore 1–4 were weakly positive, 10 patients (20%) with Artemis protein immunoscore 5–7 were moderately positive, and 4 patients (8%) with Artemis protein immunoscore 8–12 were highly positive (Figure 1b). Furthermore, no significant differences in clinical features, such as sex, age, tumor location, clinical T stage, and clinical N stage, were observed between the two groups (*p* > 0.05) (Table 1).

### 3.2. MRI Tumor Regression Grade of Primary Tumors after Chemoradiotherapy and Its Association with Artemis Expression

All 50 patients underwent rectal MRI examination before chemoradiotherapy, but only 47 patients underwent MRI examination after chemoradiotherapy. Three patients were evaluated with CT after chemoradiotherapy. Of these 47 patients, 41 underwent an MRI examination 4–8 weeks after radiotherapy and 6 underwent an MRI examination 1–2 weeks after radiotherapy. The median time from the end of radiotherapy to MRI examination was 6.5 weeks (range 1–8).

**Figure 2:**
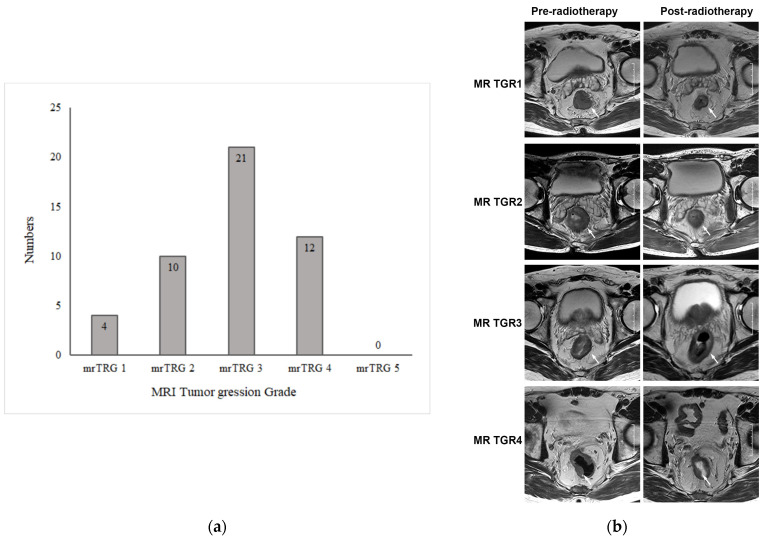
MRI tumor regression grade of primary tumors after chemoradiotherapy. (**a**) MRI tumor regression grade (mrTRG) of 50 patients with rectal cancer after neoadjuvant chemoradiotherapy. (**b**) Representative images of MRI tumor regression grade (mrTRG) of primary tumor after neoadjuvant chemoradiotherapy. The images in the left column are axial T2-weighted MR images before treatment, showing an intermediate-signal-intensity rectal mass. The images in the right column are axial T2-weighted MR images after treatment, showing that the rectal mass had shrunk to varying degrees. The white arrows indicate rectal lesions.

The MRI results pre- and post radiotherapy were independently reviewed by two experienced abdominal radiologists. The tumor response to preoperative chemoradiotherapy was evaluated using mrTRG. For inconsistent results, a consensus was reached through discussion. The mrTRG results are shown in Figure 2a. Among 47 patients, 4 achieved a radiological complete response and were considered as mrTRG1, 10 were mrTRG2, 21 were mrTRG3, and 12 were mrTRG4. None of them was mrTRG5. The representative image of mrTGR is shown in Figure 2b.

The correlations between mrTGR and immunoscore for Artemis expression were evaluated to understand the internal association between Artemis expression and mrTGR of the 47 patients. The relevant data of the 47 patients are shown in Table 2. Our results suggested that mrTGR positively correlated with the immunoscore of Artemis protein in the 47 patients (*r* = 0.304, *p* = 0.038). This means that mrTRG was higher in patients with high Artemis expression who underwent preoperative chemoradiotherapy than in those with low Artemis expression.

### 3.3. RCRG of Primary Tumor after Chemoradiotherapy and Its Association with Artemis Expression

All patients underwent transabdominal laparoscopic rectal resection according to the total mesorectal excision principle. Of the 50 patients, 43 underwent surgery 6–9 weeks after chemoradiotherapy and 4 underwent surgery within 6 weeks after chemoradiotherapy. Three patients underwent surgery more than 9 weeks after chemoradiotherapy. The median time from the end of radiotherapy to surgery was 7 weeks (range 4–14 weeks).

**Figure 3:**
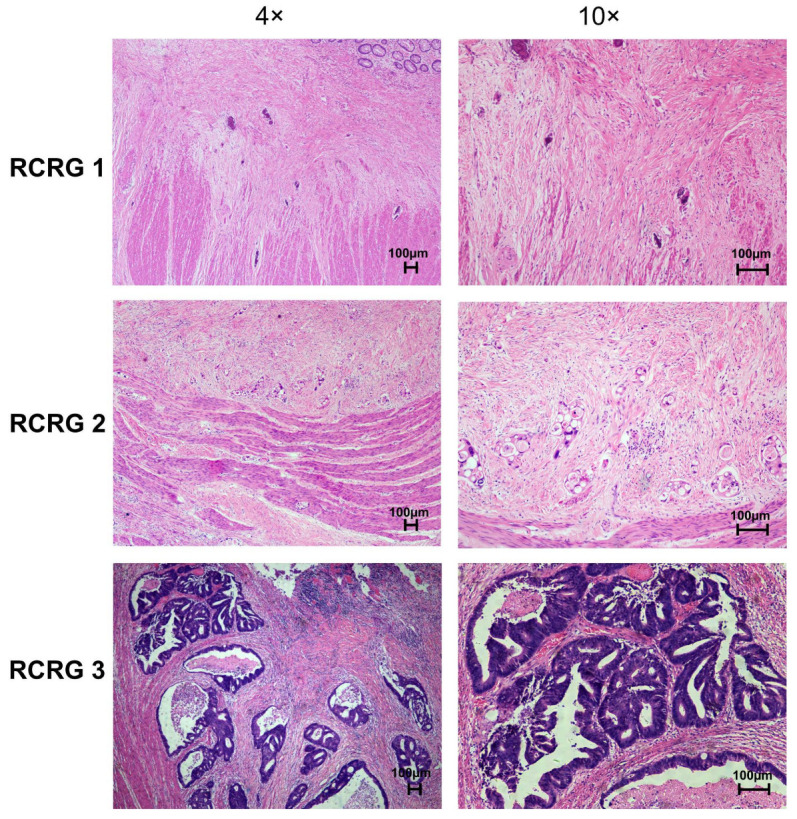
Representative images of pathological rectal cancer regression grade (RCRG) of primary rectal tumor tissues after preoperative chemoradiotherapy (H&E stain).

All resection specimens were examined by the pathologists. The regression of primary tumors was determined according to Wheeler’s RCRG criteria. The representative image of RCRG results is shown in Figure 3. Among 50 patients, 17 (34%) showed a “good” response to preoperative chemoradiotherapy (RCRG1), whereas 4 patients (8%) demonstrated a “poor” response (RCRG3). The remaining 29 patients (58%) showed marked fibrosis but with macroscopic tumors still present (RCRG2) (Table 3).

We further examined the correlations between RCRG and Artemis expression of the 50 patients with rectal cancer. The association of RCRG with Artemis expression is listed in Table 4. A positive correlation was found between RCRG and immunoscore for Artemis protein (*r* = 0.387, *p* = 0.005). In other words, the high Artemis immunoscore was correlated with the high RCRG score. We further divided these patients into two groups: response group (RCRG1 + RCRG2) and non-response group (RCRG3). Correlation analysis shows that there is a significant negative correlation between high Artemis immunoscore and treatment response (*r* = −0.532, *p* < 0.001) (Table 5). These results imply that high Artemis expression was associated with poor treatment response.

### 3.4. Pretreatment Artemis Expression Status Is Not Correlated with Overall Survival of Patients

**Figure 4:**
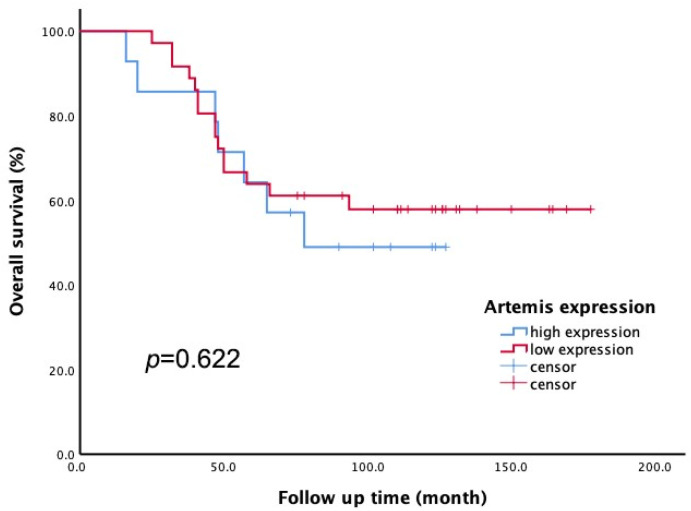
The Kaplan–Meier method was applied to assess overall survival (OS). There was no significant difference in 5-year OS rate between patients with low Artemis expression and those with high Artemis expression (*p* = 0.622).

All 50 enrolled patients completed subsequent follow-up to obtain overall survival (OS) data. According to the Artemis immunoscore, the 50 patients were divided into two groups: low Artemis expression (score 1–4) and high Artemis expression (score 5–12). Among them, 36 patients had low Artemis expression, while 14 patients had high Artemis expression. The impact of pretreatment Artemis expression on OS was evaluated using Kaplan–Meier survival analysis. After a median follow-up of 7.5 years (range, 1.4–13.8 years), the 5-year OS rates were found to be 63.9% in the low-Artemis-expression group and 64.3% in the high-Artemis-expression group. However, there was no significant difference in the 5-year OS between the high- and low-Artemis-expression groups (*p* = 0.622; see Figure 4).

## 4. Discussion

Although preoperative chemoradiotherapy has become the standard treatment regimen for LARC, it does not necessarily mean that every patient can benefit from it. Therefore, it is significant to screen patients who are resistant to radiotherapy to avoid unnecessary treatment. Extensive research has been performed to identify biological markers that can predict a successful response to radiation, translating into improved survival for the personalized treatment of rectal cancer. Recent studies indicate that some NHEJ members have a predictive value for the response to neoadjuvant treatment and can serve as a novel therapeutic target in rectal cancer [11,23,24,25]. As a major member of NHEJ, the heterodimer Ku70/80 plays a critical role in DNA repair. Pucci et al. observed that the expression of Ku70/80 intensity was slightly lower in responders than in non-responders, indicating a potential role of Ku70/80 as a new predictor of the response to preoperative chemoradiotherapy [23]. Artemis is also an important member of NHEJ and serves as a multifunctional protein in DSB repair via all major repair pathways [26]. Our study found that a higher Artemis expression in rectal cancer tissues was significantly correlated with a poor tumor response to preoperative chemoradiotherapy.

In our previous studies, we compared the differences in Artemis expression levels and radiosensitivity between colorectal cancer cell lines RKO and HCT116. We found that the expression level of Artemis was higher in RKO cells than in HCT116 cells. Correspondingly, the colony-forming assays showed that the RKO cells were more radioresistant than HCT116 cells. Furthermore, we found that the knockdown of Artemis in human colorectal cancer cells enhanced the sensitivity of cancer cells to DNA-damaging agents including radiation. In this study, we retrospectively investigated the clinical data of 50 patients with rectal cancer and found that the tumor response to preoperative chemoradiotherapy was positively correlated with the expression level of Artemis in rectal cancer tissues. The consistency between preclinical and clinical research results indicated that rectal cancer cells overexpressing Artemis exhibited a more radioresistant phenotype, thereby affecting the outcome of treatment.

In this study, 86% of patients underwent surgical treatment 6–9 weeks after chemoradiotherapy. Therefore, the time for evaluating the pathologic response of tumors was also 6–9 weeks after chemoradiotherapy. This was consistent with the current clinical guidelines that recommended the optimal time interval between the completion of chemoradiotherapy and surgery as 6–8 weeks [27]. The Lyon trial demonstrated that a 6-week delay significantly increased the pCR rate (26% vs. 10%) compared with a 2-week interval [28]. However, the GRECCAR6 trial found that waiting for 11 weeks after RCT did not increase the pCR rate (17.4% vs. 15%) and might be associated with higher morbidity and more difficult surgical resection [29]. Therefore, it was appropriate to analyze the tumor pathological tissue response 6–8 weeks after CRT. This study showed that 34% of patients achieved RCRG1, which included PCR and a near-complete pathologic response (persistence of some tumor cells). This result is consistent with previous findings.

We also used MRI to evaluate the tumor response to treatment. MRI is widely used in evaluating rectal cancer with the advantages of superior soft-tissue contrast, multiplanar imaging, and functional evaluation. MRI can accurately evaluate primary tumor staging based on the depth of invasion within and through the rectal wall. It can also evaluate morphologic components within the tumors after preoperative chemoradiotherapy, including fibrosis and mucinous changes. These features have been shown to correlate well with the pathologic response [21]. The MRI-based tumor regression grade (mrTRG) has important prognostic implications that have been shown to correlate with the 5-year disease-free survival rate [30,31]. In this study, the time of MRI examination was mainly 1–2 weeks before surgery, which was consistent with the time of pathologic response evaluation. This study found that, similar to pathological RCRG, mrTGR was also positively correlated with the expression of Artemis. This further provided evidence for the correlation between the tumor response to treatment and the expression level of Artemis in rectal cancer cells.

In this study, we also assessed the influence of Artemis expression on the clinical outcome of patients. Our results indicated that the pretreatment Artemis immunoscore status of the tumor tissue had no effect on the 5-year OS rate in locally advanced rectal cancer patients. One of the reasons is due to sample size limitations. Another possible reason is that we only assessed Artemis expression in the primary tumor and did not evaluate its expression in lymph nodes, because of the difficulty of obtaining biopsy tissue from pelvic lymph nodes. Previous studies indicated that the response of primary tumors and metastatic lymph nodes to radiotherapy might differ. In fact, 10% of patients with ypT0 tumors had positive nodes after chemoradiotherapy and resection [32]. The lymph node status after preoperative chemoradiotherapy was also a crucial factor affecting the prognosis of patients. Thirdly, in this study, all patients underwent the same concurrent chemoradiotherapy regimen, which consisted of the combination of radiotherapy with capecitabine monotherapy. This regimen is considered one of the standard approaches for preoperative chemoradiotherapy in locally advanced rectal cancer. There is also insufficient evidence to suggest that the addition of oxaliplatin or molecular target agents can improve tumor response or patient survival [33,34]. The use of the same treatment regimen helps minimize the impact of different chemotherapy drugs on the response to preoperative treatment. However, it should be noted that overall survival can also be influenced by the treatment regimens that patients receive subsequently. Due to incomplete information regarding the subsequent treatments of some patients, further analysis on this matter is not feasible.

This study had some potential limitations. First, the retrospective nature of this study led to selection bias in this study. Second, the small number of cases led to an imbalanced grouping. Third, we were not sure whether irradiation affected the expression level of Artemis in cells, thus influencing the accuracy of Artemis as a predictive biomarker. We will collect more cases and detect the Artemis expression in tissue after CRT in the future. Fourth, the clinical information of patients was incomplete due to some patients not receiving further treatment at our hospital. This limitation hindered us from conducting further analysis on the correlation between Artemis expression and disease-free survival (DFS) in patients. We need to conduct a prospective trial to validate and confirm our conclusions.

## 5. Conclusions

In this study, our results indicated that the expression level of Artemis in rectal cancer biopsy tissue before treatment was correlated with the tumor response to preoperative chemoradiotherapy. This indicated that we could predict the tumor response to radiotherapy by detecting Artemis expression in biopsy tissues of patients with rectal cancer. This was the first report on Artemis expression in a biopsy specimen as a predictor of the tumor response to preoperative chemoradiotherapy in patients with LARC. As the role of Artemis in NHEJ becomes more widely known, there have been studies exploring the inhibition of Artemis nuclease activity as a new strategy for tumor treatment [35,36]. Our result might not only help screen out patients who are not sensitive to radiotherapy but also promote the design of new chemo-radiosensitizers targeting Artemis to increase the success of neoadjuvant therapy.

## Figures and Tables

**Table 1:** Clinicopathological characteristics of patients and Artemis expression.

Clinical Characteristic	Total (*n* = 50)	Immunoscore of Artemis			*p* Value
		1–4	5–7	≥8	
Sex					
Female	13	9	3	1	0.871
Male	37	27	7	3	
Age (year)					
>60	21	14	5	2	0.805
≤60	29	22	5	2	
Location (distance to the anus)					
≤5 cm	32	25	3	3	0.064
>5 cm	18	11	7	1	
Clinical (T) stage					
T2–3	43	32	7	4	0.216
T4	7	4	3	0	
Clinical (N) stage					
N0–1	38	26	8	4	0.741
N2	12	10	2	0	

**Table 2:** Correlation between the immunoscore of Artemis expression and the mrTRG in 50 patients.

Immunoscore of Artemis	Total (*n* = 47)	mrTRG1	mrTRG2	mrTRG3	mrTRG4	mrTRG5	*r*/*p*
1–4	34	3	9	16	6	0	0.304/0.038
5–7	9	1	1	4	3	0	
8–12	4	0	0	1	3	0	

Abbreviations: mrTGR, MRI tumor regression grade; *r*/*p*, correlation coefficient/*p* value.

**Table 3:** RCRG with respect to primary tumors in 50 patients treated with preoperative chemoradiotherapy and surgery.

RCRG	Histological Features	No. of Patients (%)
1	Sterilization or only microscopic foci of adenocarcinoma remaining, with marked fibrosis	17 (34)
2	Marked fibrosis but macroscopic disease present	29 (58)
3	Little or no fibrosis, with abundant macroscopic disease	4 (8)

Abbreviations: RCRG, rectal cancer regression grade.

**Table 4:** Correlation between the immunoscore of Artemis expression and RCRG.

Immunoscore of Artemis	Total (*n* = 50)	RCRG 1	RCRG 2	RCRG 3	*r*/*p*
1–4	36	14	22	0	0.387/0.005
5–7	10	2	6	2	
8–12	4	0	2	2	

Abbreviations: RCRG, rectal cancer regression grade; *r*/*p*, correlation coefficient/*p* value.

**Table 5:** Correlation between the immunoscore of Artemis expression and treatment response.

Immunoscore of Artemis	Total (*n* = 50)	Response	Non-Response	*r*/*p*
1–4	36	36	0	−0.532/<0.001
5–7	10	8	2	
8–12	4	2	2	

Abbreviations: *r*/*p*, correlation coefficient/*p* value.

## Data Availability

The data presented in this study are available on request from the corresponding author.
